# Durian fruit pulp extract enhances intracellular glutathione levels, mitigating oxidative stress and inflammation for neuroprotection

**DOI:** 10.1038/s41598-024-65219-6

**Published:** 2024-07-02

**Authors:** Gholamreza Khaksar, Su Lwin Lwin Myint, Hasriadi, Pasarapa Towiwat, Supaart Sirikantaramas, Ratchanee Rodsiri

**Affiliations:** 1 Center of Excellence in Molecular Crop, Department of Biochemistry, Faculty of Science, Chulalongkorn University, 254 Phayathai Road, Bangkok, 10330 Thailand; 2 Preclinical Toxicity and Efficacy Assessment of Medicines and Chemicals Research Unit, Chulalongkorn University, Bangkok, 10330 Thailand; 3 Animal Models of Chronic Inflammation-Associated Diseases for Drug Discovery Research Unit, Chulalongkorn University, Bangkok, 10330 Thailand; 4 Department of Pharmacology and Physiology, Faculty of Pharmaceutical Sciences, Chulalongkorn University, Bangkok, 10330 Thailand; 5 Omics Sciences and Bioinformatics Center, Chulalongkorn University, 254 Phayathai Road, Bangkok, 10330 Thailand

**Keywords:** Biotechnology, Chemical biology

## Abstract

Durian (*Durio zibethinus* L.) fruit pulp is a rich source of γ-glutamylcysteine (γ-EC), a direct precursor to the antioxidant glutathione (GSH). This study elucidated the in vitro neuroprotective potential of unripe durian fruit pulp extract (UDE) against H_2_O_2_-induced neurotoxicity in SH-SY5Y cells and neuroinflammation in lipopolysaccharide (LPS)-stimulated BV-2 cells. Treatments with γ-EC, GSH standards, or UDE exhibited no cytotoxicity in SH-SY5Y and BV-2 cells, except at high concentrations. A 4-h pretreatment with 100 µM γ-EC or UDE containing 100 µM γ-EC significantly increased SH-SY5Y cell viability post H_2_O_2_ induction. Moreover, a similar pretreatment reduced LPS-stimulated production of proinflammatory cytokines in BV-2 cells. The neuroprotective effect of UDE is primarily attributed to γ-EC provision and the promotion of GSH synthesis, which in turn elevates intracellular GSH levels and reduces proinflammatory cytokines. This study identifies γ-EC in UDE as a potential neuroprotective biomarker boosting intracellular GSH levels, providing insights into UDE's therapeutic potential.

## Introduction

Neurodegenerative diseases, such as Parkinson’s (PD) and Alzheimer’s (AD), are largely due to neuronal damage often triggered by oxidative stress^1,2^. Hydrogen peroxide (H_2_O_2_), a naturally occurring reactive oxygen species (ROS), is seen as a harmful molecule involved in the onset of these diseases. It adds to oxidative stress, triggers apoptosis, and furthers the pathological progression of these conditions^3^. ROS exert their harmful effects by deactivating enzymes, oxidizing proteins, damaging DNA, initiating lipid peroxidation, and denaturing proteins. This cellular disruption compromises cell function and integrity, leading to neuronal injury, necrosis, and apoptosis, thereby contributing to neurodegenerative diseases and related functional impairments^4,5^. Mitochondria are the primary ROS producers. The noticeable increase in ROS accumulation is particularly evident as the aging process unfolds^6^. As a result, controlling oxidative stress has become a key neuroprotective strategy.

Glutathione (GSH), a vital endogenous antioxidant, is the most abundant low-molecular-weight thiol in mammalian cells. It plays a key role in preserving and regulating the thiol-redox status of cells. Besides its function as a reducing agent and primary antioxidant^7^, GSH participates in several critical physiological processes, including cell proliferation, apoptosis, immune function, and fibrogenesis^8^. Within cells, GSH primarily exists in a reduced state, along with two interchangeable oxidized forms: the disulfide form (GSSG) and a mixed disulfide with protein thiols (GSSR). The de novo synthesis of GSH takes place in the cytosol of human cells and involves two sequential ATP-dependent enzymatic reactions^9^. The first and rate-limiting step involves generating the dipeptide γ-glutamylcysteine (γ-EC) from glutamate and cysteine, catalyzed by glutamate cysteine ligase (GCL). The second step involves adding glycine to γ-EC to form GSH, catalyzed by glutathione synthetase (GSS)^10^. Given the health-promoting functions of GSH, increasing intracellular GSH concentration under oxidative stress (maintaining homeostasis of reduced/oxidized ratios) is crucial for protecting against cell injury. However, GSH levels tend to decrease with aging and in various age-related neurodegenerative diseases, especially Alzheimer’s disease, the most prevalent form of dementia affecting the elderly^11–13^. Cellular GSH depletion often occurs because of the downregulation of expression or a decrease in the specific activities of the first biosynthetic enzyme GCL^14^.

GSH faces limitations as an effective therapeutic agent, with exogenous supplementation showing minimal impact on intracellular GSH levels or oxidative stress markers. This holds true for both single-dose administration^15,16^ and longer-term supplementation^17^. Notably, in vivo experiments indicate that γ-GC demonstrates superior therapeutic effects against oxidative stress and inflammation compared to GSH^18^. The existing body of literature underscores the challenges associated with GSH's poor bioavailability, attributed to its unfavorable biochemical and pharmacokinetic properties^10,16^.

There is a concept suggesting that the availability of γ-EC could become a limiting factor in maintaining necessary cellular GSH levels to efficiently protect against oxidative stress and potential physiological damage. The ability of γ-EC to enhance cellular GSH levels was first documented by Anderson and Meister^10^ in a rat model. Subsequent studies have shown that γ-EC administration can increase intracellular GSH levels and reduce oxidative damage in mice^18–21^, human cell lines^19,20,22,23^ and humans^24^. In addition to its therapeutic role in increasing intracellular GSH levels, γ-EC also exhibits significant antioxidant properties^20,25^. It also acts as a vital cofactor for the antioxidant enzyme glutathione peroxidase (GPx)^20^.

Bioactive compounds in many herbal medicines have been validated for their neuroprotective effects, as demonstrated by their ability to neutralize free radicals. This process helps rejuvenate nerve cells, protecting them from oxidative stress-induced damage. Durian (*Durio zibethinus* L.), a crucial tropical fruit crop, holds significant economic value in Southeast Asia. Durian pulp is known for its high concentration of essential vitamins and minerals. Another significant nutritional aspect of durian pulp is its outstanding antioxidant properties, which are primarily attributed to its high polyphenol content^26^. Among the bioactive compounds in durian fruit pulp, sulfur-containing metabolites like GSH and its precursor γ-EC are of considerable interest for human consumption. Durian fruit pulp is a rich natural source of GSH and γ-EC^27,28^, with about 2.6 and 14 mg/g on a dry weight basis, respectively^29^. Notably, the levels of GSH and γ-EC in ripe durian pulp significantly exceed those found in various fruits and vegetables^29^. Given this nutritional profile, developing durian pulp extract as a dietary supplement offers a promising opportunity to meet the recommended daily intake of GSH and γ-EC, while also leveraging its other potential health benefits.

Past research has highlighted the medicinal properties of durian fruit pulp extract, including its antiproliferative effects on various human cancer cell lines^30–34^, neuroprotective effect on the human neuroblastoma cell line SH-SY5Y^35^, and antidiabetic effect on the HepG2 cell line by regulating glycolysis regulatory genes^32^. However, to our knowledge, no studies have explored the impact of durian pulp extract on boosting intracellular GSH levels in human cells under oxidative stress. In this study, we assessed the neuroprotective effect of unripe durian fruit pulp extract (UDE), rich in γ-EC and without sulfur-containing volatiles, against H_2_O_2_-induced neurotoxicity in SH-SY5Y cells. We also examined its role in inhibiting neuroinflammation by downregulating proinflammatory cytokines in lipopolysaccharide (LPS)-stimulated BV-2 cells. In this study, we standardized the UDE based on the γ-EC content, considering its potential advantages over GSH in the context of therapeutic effects. Our results suggest that UDE, a natural supplement, can offer protection against neurodegeneration and neuroinflammation. Importantly, this is the first study to identify γ-EC in durian pulp extract as a potential neuroprotective biomarker.

## Results

### Effect of γ-EC and GSH standards and UDE on SH-SY5Y cell viability

**Figure 1:**
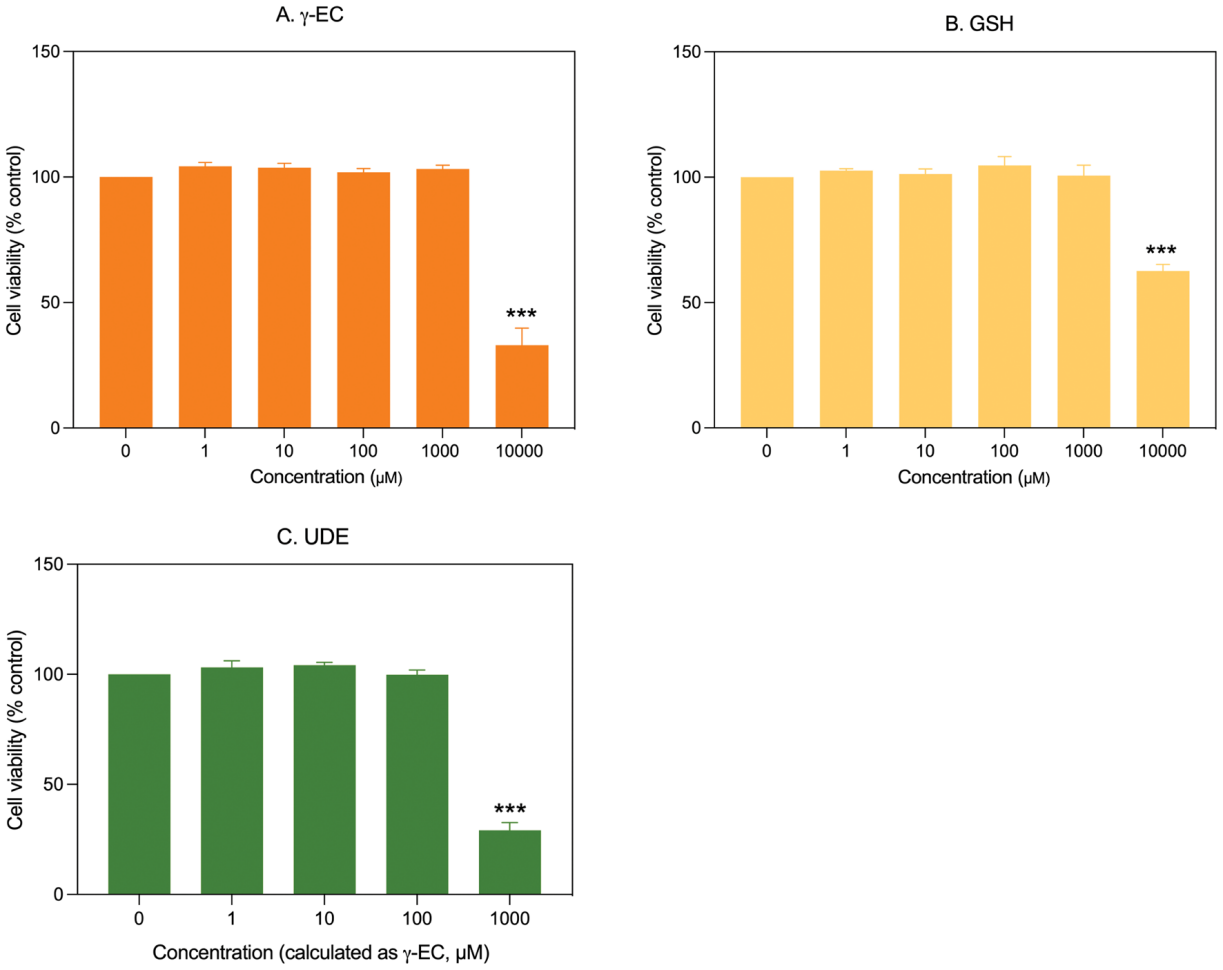
Impact of γ-glutamylcysteine (γ-EC) and glutathione (GSH) standards and the unripe durian fruit pulp extract (UDE) on SH-SY5Y cell viability. The in vitro cytotoxicity of varying concentrations of γ-EC (**A**) and GSH (**B**) standards and the UDE containing different concentrations of γ-EC (**C**) were evaluated. Data are presented as the mean ± standard error of the mean (SEM) (n = 3). ***p < 0.001 vs control (0 µM).

The cytotoxicity of γ-EC and GSH standards, as well as UDE, was evaluated in vitro against SH-SY5Y cells using a resazurin assay. The application of 1, 10, 100, and 1000 µM of γ-EC or GSH standards did not exhibit any harmful effect on cell viability compared to the control. However, a significant decrease in cell viability was observed when these compounds were used at 10,000 µM, indicating their cytotoxic effect. For UDE, no toxic effect was observed for extracts containing 1, 10, and 100 µM γ-EC. However, a significant reduction in cell viability was noted for UDE containing 1000 µM γ-EC (Fig. 1).

### *γ-*EC and GSH standards protected SH-SY5Y cells against H_2_O_2_-induced injury

**Figure 2:**
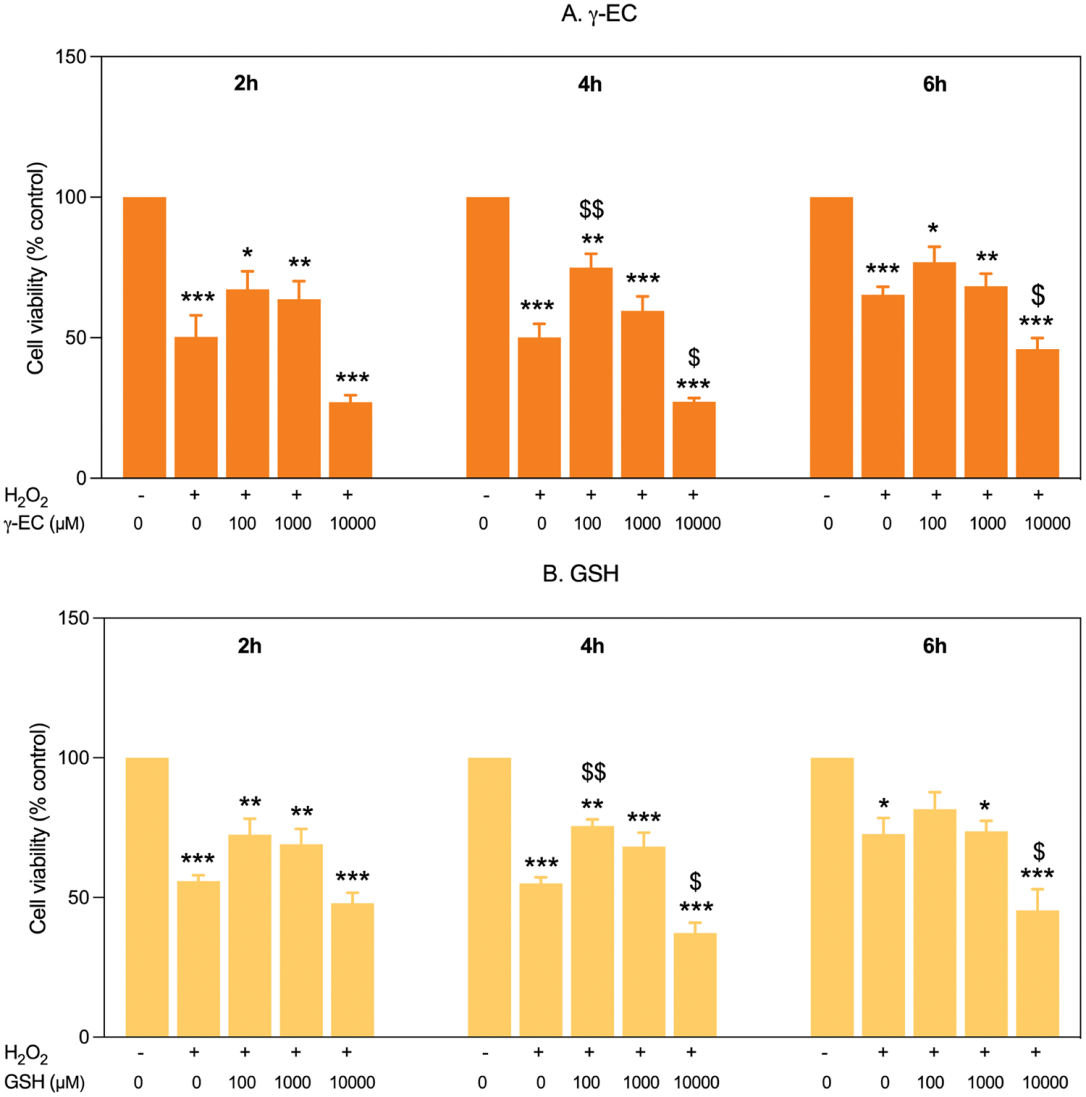
Effect of γ-glutamylcysteine (γ-EC) and glutathione (GSH) standards on SH-SY5Y cell viability under H_2_O_2_-induced stress. The protective effects of different concentrations (100, 1000, and 10,000 µM) of γ-EC (**A**) and GSH (**B**) standards at 2, 4, and 6 h pretreatment periods against H_2_O_2_-induced oxidative damage on SH-SY5Y cells were studied. Data are expressed as the mean ± standard error of the mean (SEM) (n = 3). *p < 0.05, **p < 0.005, ***p < 0.001 vs 0 µM without H_2_O_2_; ^$^*p* < 0.05, ^$$^*p* < 0.005 vs H_2_O_2_-induced cells without pretreatment with the compounds.

The protective effects of γ-EC and GSH standards against H_2_O_2_-induced toxicity in SH-SY5Y cells were evaluated in vitro. The cells were incubated with varying concentrations (100, 1000, and 10,000 µM) of the compounds for 2, 4, and 6 h. Following incubation, the cells were exposed to H_2_O_2_. As shown in Fig. 2, the application of the compounds at 100 and 1000 µM for 2 h did not protect the cells against H_2_O_2_-induced toxicity, as there was no significant difference in cell viability compared to the H_2_O_2_-treated cells without the compound. However, a significant increase in cell viability was observed for γ-EC (100 µM)- and GSH (100 µM)-pretreated cells after a 4 h incubation period, suggesting the protective effect of the compounds. For the cells incubated for 6 h, a similar observation to the 2 h group was found. Notably, the application of the compounds at 10,000 µM significantly reduced cell viability compared with the H_2_O_2_-treated cells without the compound (Fig. 2), suggesting its cytotoxicity as previously observed (Fig. 1). These observations suggest that a 4 h incubation period with 100 µM γ-EC or GSH standards is the optimal condition. The protective effect of a 4 h pretreatment with UDE against H_2_O_2_-induced toxicity was then evaluated.

### UDE protected SH-SY5Y cells against H_2_O_2_-induced toxicity

**Figure 3:**
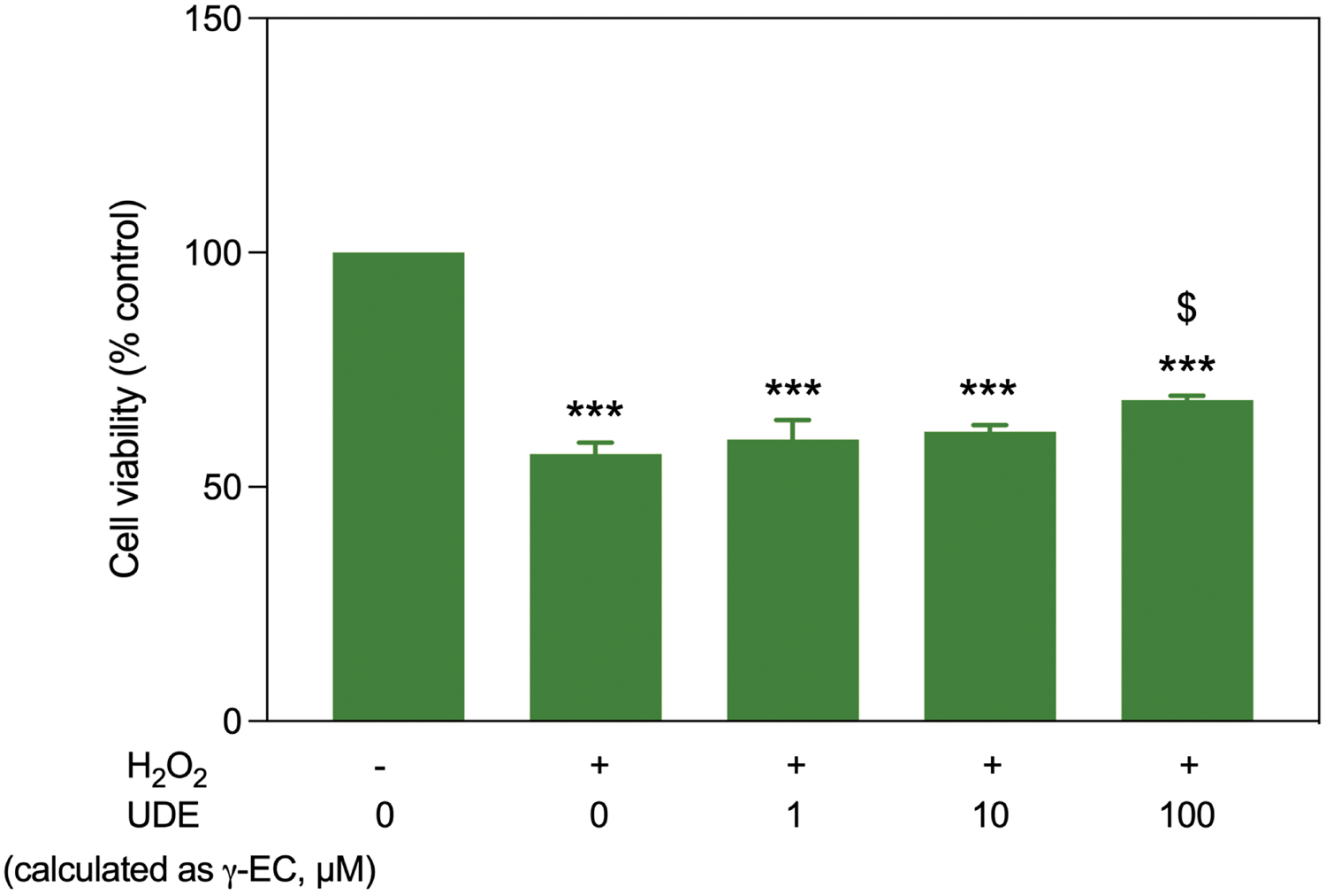
Impact of unripe durian fruit pulp extract (UDE) against H_2_O_2_-induced toxicity on SH-SY5Y cells. The protective effect of a 4 h pretreatment with UDE containing 1, 10, and 100 µM γ-EC against H_2_O_2_-induced oxidative damage on SH-SY5Y cells was evaluated. Data are expressed as the mean ± standard error of the mean (SEM) (n = 3). ***p < 0.001 vs 0 µM without H_2_O_2_; ^$^*p* < 0.05 vs H_2_O_2_-induced cells without pretreatment with the UDE.

As previously observed, UDE containing 1000 µM γ-EC exhibited cytotoxicity (Fig. 1). To investigate the effect of UDE against H_2_O_2_-induced toxicity, cells were incubated with UDE containing 1, 10, and 100 µM γ-EC for 4 h, followed by H_2_O_2_ induction. For cells pretreated with 1 and 10 µM γ-EC-containing UDE, no significant difference in cell viability was observed compared to the H_2_O_2_-injured cells without pretreatment (Fig. 3). However, cells pretreated with UDE containing100 µM γ-EC showed a significant increase in cell viability, suggesting that UDE has a protective effect against oxidative stress.

### γ-EC and GSH standards and UDE enhanced SH-SY5Y intracellular GSH content

**Figure 4:**
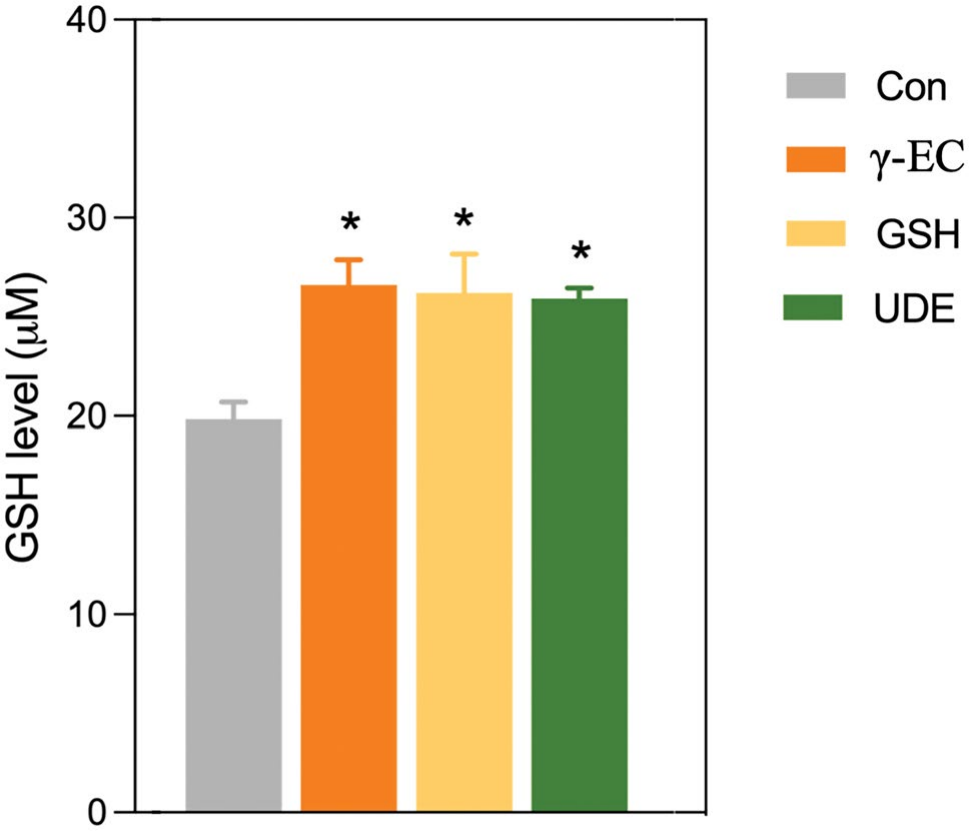
Effect of γ-glutamylcysteine (γ-EC) and glutathione (GSH) standards and the unripe durian fruit pulp extract (UDE) on intracellular GSH content of SH-SY5Y cells. The levels of intracellular GSH were measured following a 4 h incubation with 100 µM of γ-EC, GSH, or UDE containing 100 µM γ-EC. Data are expressed as the mean ± standard error of the mean (SEM) (n = 3). *p < 0.05 vs Con (control; cells without incubation).

In our previous investigation, we identified the protective effect of a 4 h incubation with 100 µM of γ-EC, GSH, or UDE containing 100 µM γ-EC against oxidative damage caused by H_2_O_2_. We then investigated whether this protection was linked to increased intracellular GSH levels. Our results showed a significant increase in intracellular GSH content in cells treated with 100 µM of γ-EC, GSH, or UDE containing 100 µM γ-EC, compared to the untreated control cells (Fig. 4).

### Effect of γ-EC and GSH standards and UDE on BV-2 cell viability

**Figure 5:**
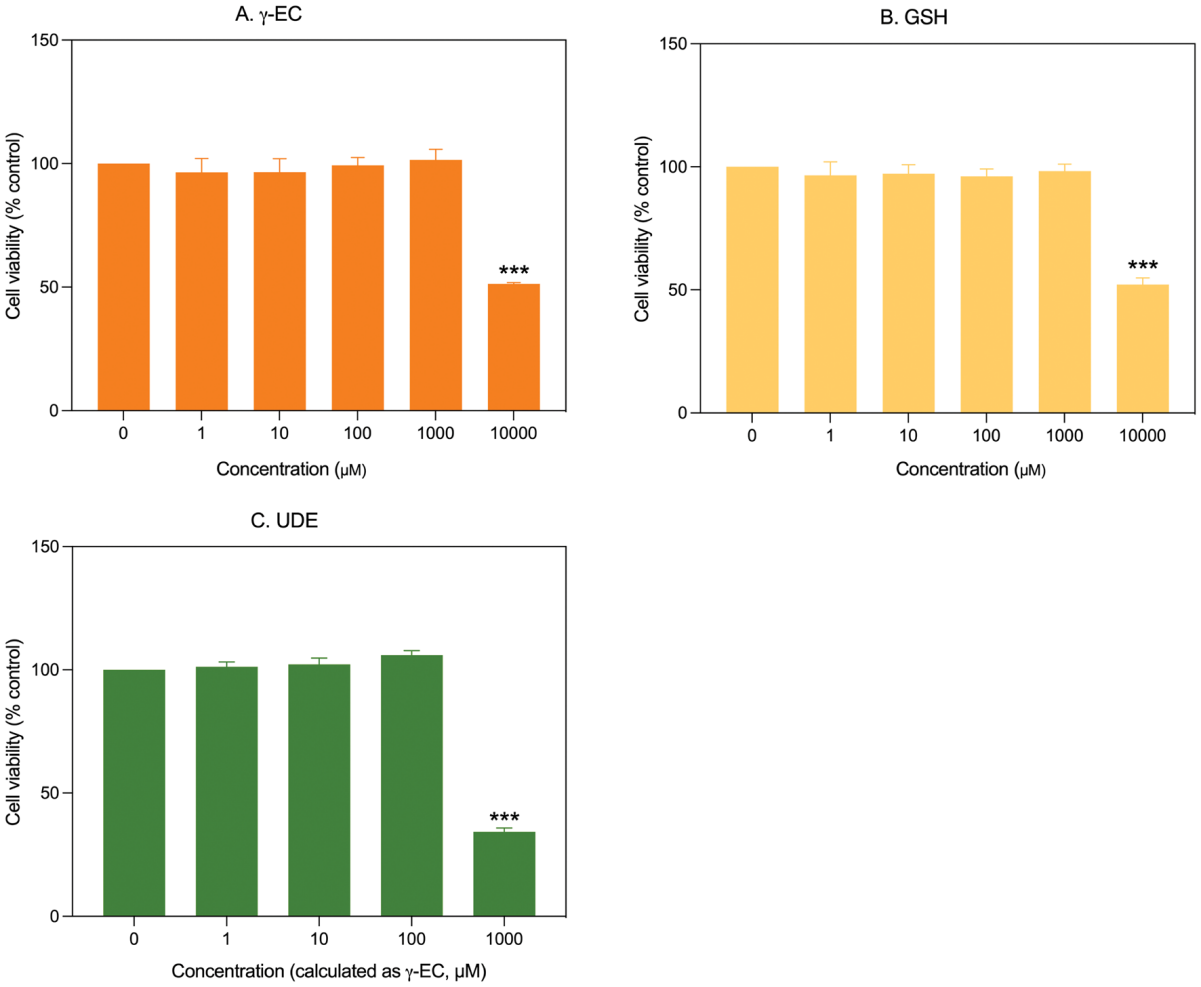
Impact of γ-glutamylcysteine (γ-EC) and glutathione (GSH) standards and the unripe durian fruit pulp extract (UDE) on BV-2 cell viability. The in vitro cytotoxicity of different concentrations of γ-EC (**A**) and GSH (**B**) standards and the UDE containing different concentrations of γ-EC (**C**) were evaluated. Data are expressed as the mean ± standard error of the mean (SEM) (n = 3). ***p < 0.001 vs control (0 µM).

In vitro cytotoxicity of γ-EC and GSH standards and the UDE at different concentrations were studied against BV-2 cells. As observed for the γ-EC and GSH standards, concentrations of 1, 10, 100, and 1000 µM showed no adverse effects on cell viability compared to the control. However, at a concentration of 10,000 µM, a notable decrease in cell viability was evident, indicating the cytotoxic effect of the compounds. For the UDE, no toxic effect was observed for 1, 10, and 100 µM γ-EC-containing UDE. However, treatment with the UDE, containing 1000 µM γ-EC, significantly reduced the cell viability (Fig. 5).

### γ-EC and GSH standards and UDE reduced LPS-stimulated proinflammatory cytokines released by BV-2 cells

**Figure 6:**
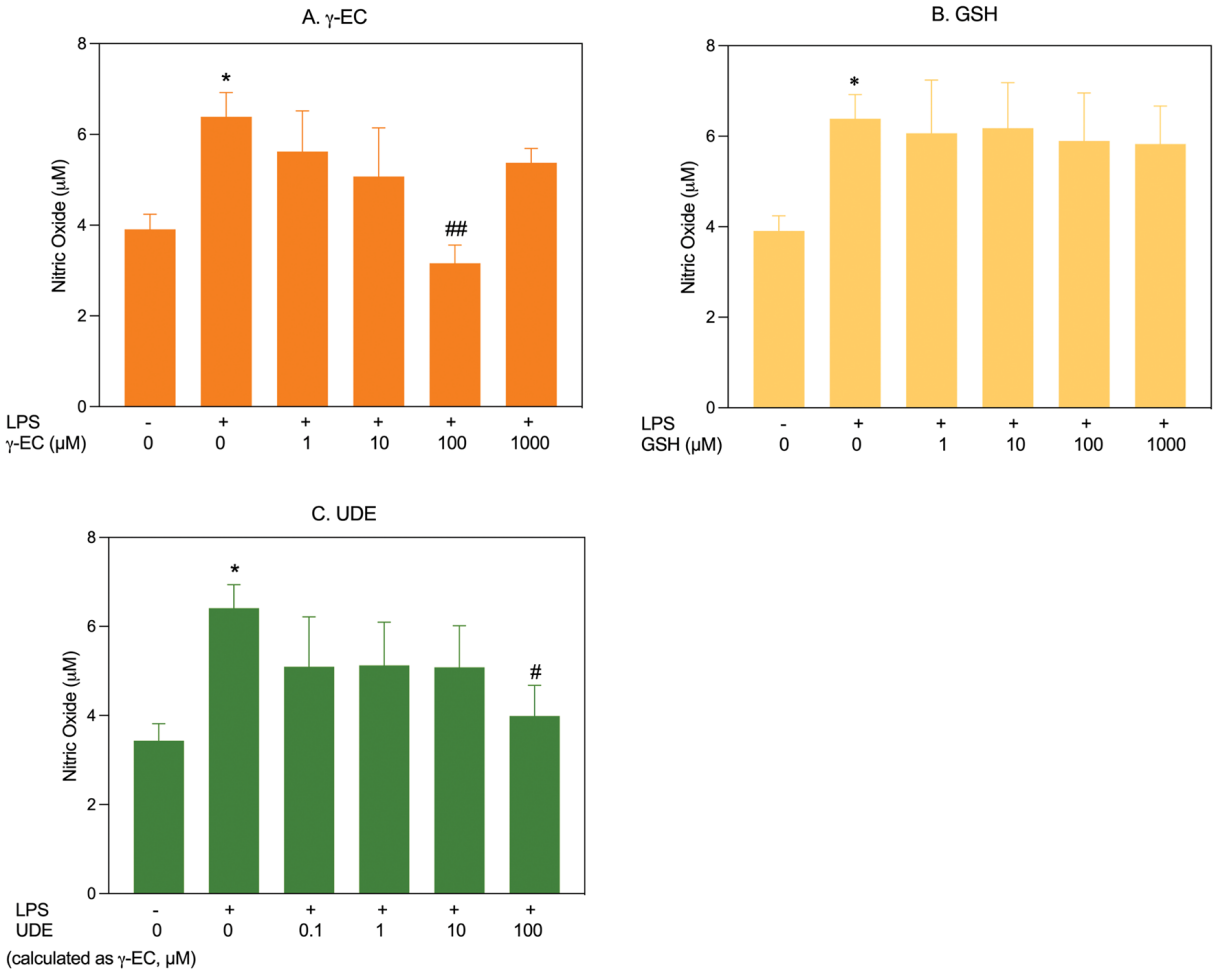
Influence of γ-glutamylcysteine (γ-EC) and glutathione (GSH) standards and the unripe durian fruit pulp extract (UDE) on nitric oxide (NO) production by BV-2 cells. The levels of a proinflammatory cytokine (NO) released by lipopolysaccharide (LPS)-stimulated BV-2 cells pretreated with different concentrations of the γ-EC (**A**), GSH (**B**), or UDE containing different concentrations of γ-EC (**C**) were measured. Data are expressed as the mean ± standard error of the mean (SEM) (n = 4). *p < 0.05 vs 0 µM without LPS; ^#^*p* < 0.05, ^##^*p* < 0.005 vs LPS-stimulated BV-2 cells without pretreatment with the compounds or the UDE.

**Figure 7:**
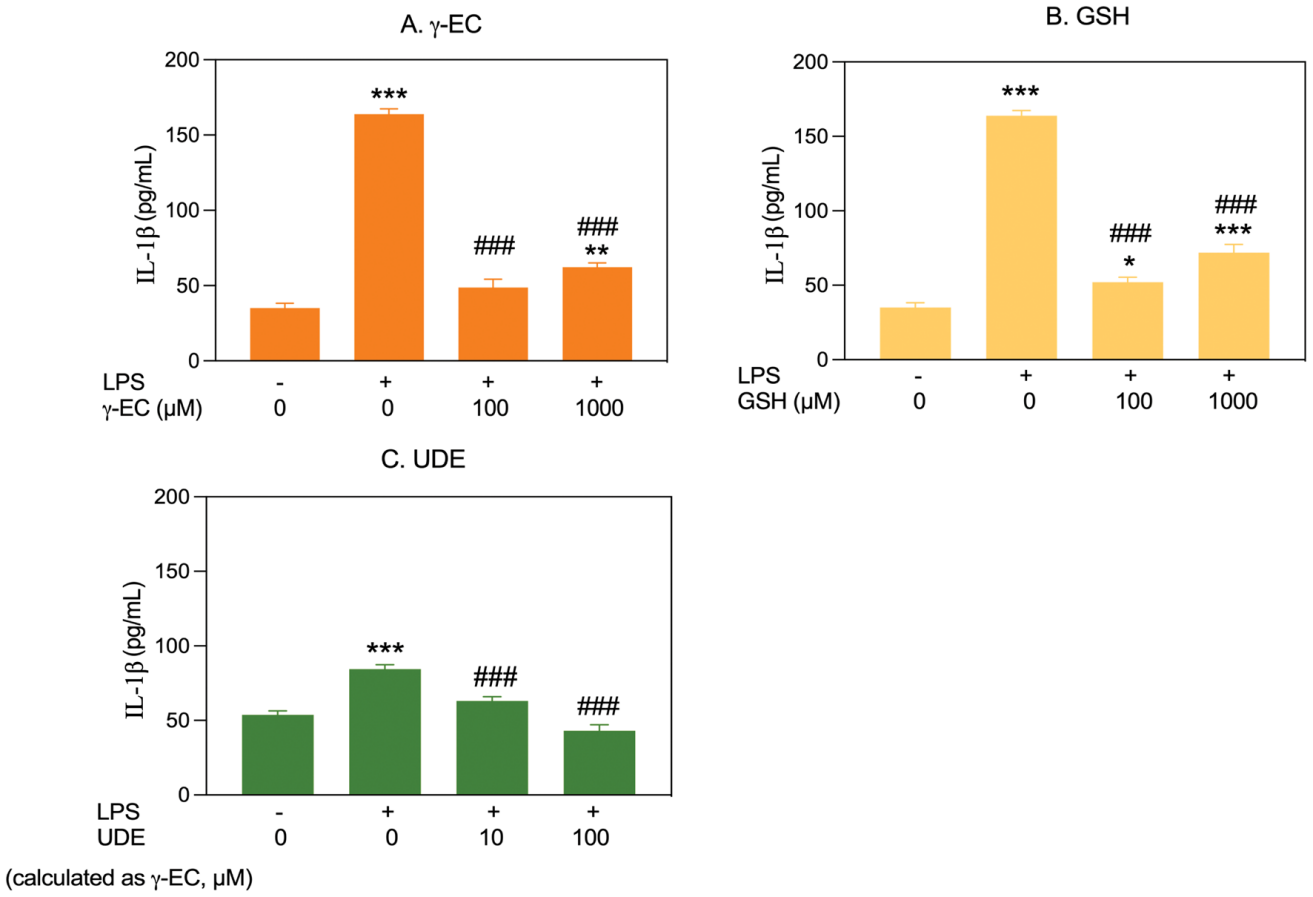
Impact of γ-glutamylcysteine (γ-EC) and glutathione (GSH) standards and the unripe durian fruit pulp extract (UDE) on interleukin (IL)-1β generation by BV-2 cells. The levels of a proinflammatory cytokine (IL-1β) released by lipopolysaccharide (LPS)-stimulated BV-2 cells pretreated with different concentrations of the γ-EC (**A**), GSH (**B**), or UDE containing different concentrations of γ-EC (**C**) were measured. Data are presented as the mean ± standard error of the mean (SEM) (n = 4). *p < 0.05, **p < 0.005, ***p < 0.001 vs 0 µM without LPS; ^###^*p* < 0.001 vs LPS-stimulated BV-2 cells without pretreatment with the compounds or the UDE.

**Figure 8:**
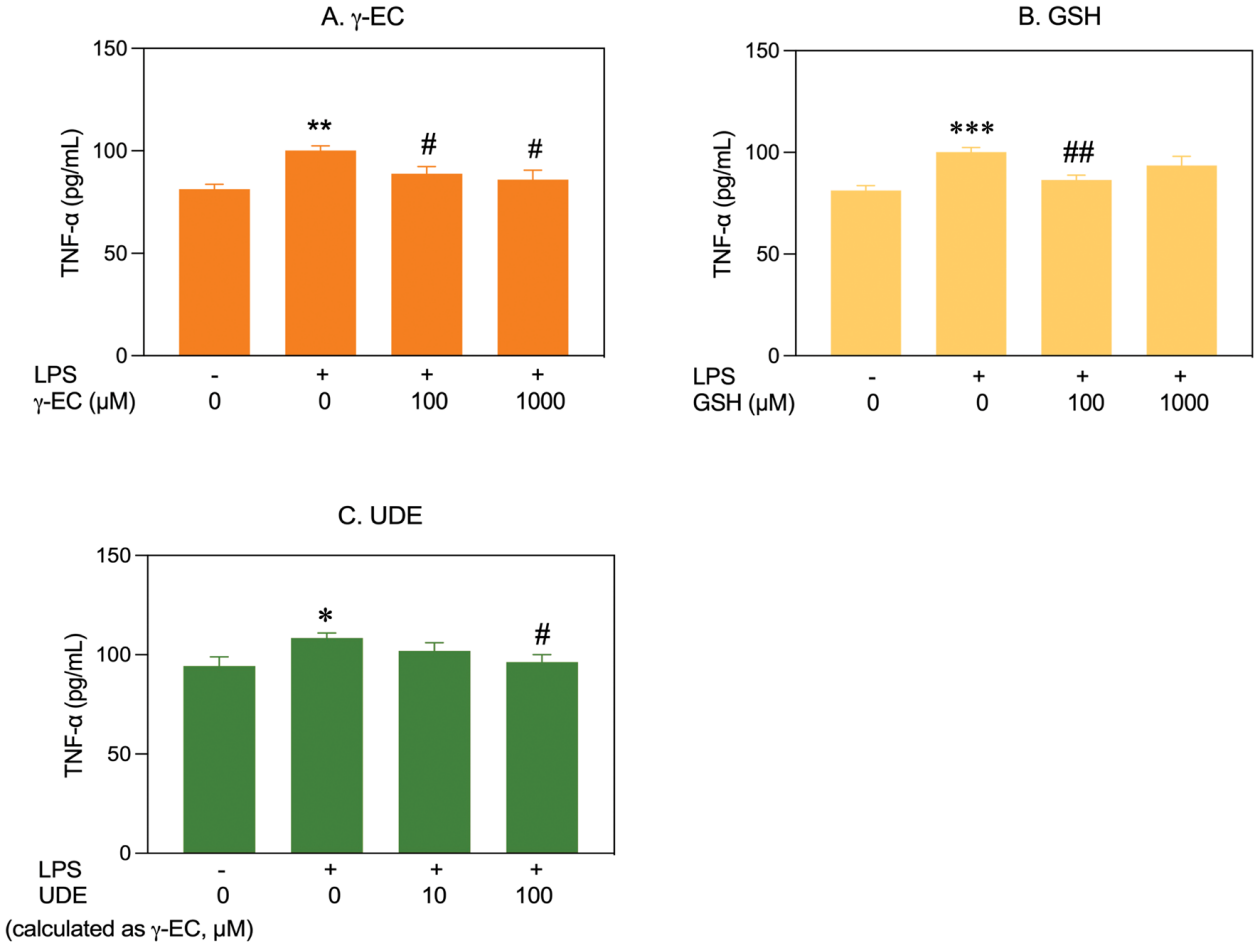
Effect of γ-glutamylcysteine (γ-EC) and glutathione (GSH) standards and the unripe durian fruit pulp extract (UDE) on tumor necrosis factor-α (TNF-α) generation by BV-2 cells. The levels of a proinflammatory cytokine (TNF-α) produced by lipopolysaccharide (LPS)-stimulated BV-2 cells pretreated with different concentrations of the γ-EC (**A**), GSH (**B**), or UDE containing different concentrations of γ-EC (**C**) were measured. Data are expressed as the mean ± standard error of the mean (SEM) (n = 4). *p < 0.05, **p < 0.005, ***p < 0.001 vs 0 µM without LPS; ^#^*p* < 0.05, ^##^*p* < 0.005 vs LPS-stimulated BV-2 cells without pretreatment with the compounds or the UDE.

This study examined the effects of pretreatment with varying concentrations of γ-EC, GSH, or UDE on proinflammatory cytokine levels (NO, IL-1β, and TNF-α) in LPS-stimulated BV-2 cells. Cell viability remained unaffected by LPS stimulation (Supplementary Fig. S1). For BV-2 cells pretreated with γ-EC (1, 10, 100, and 1000 µM), a significant reduction in LPS-induced NO production was observed specifically in cells treated with 100 µM γ-EC compared to the LPS-stimulated control cells (Fig. 6). However, GSH pretreatment showed no significant difference in NO levels across all concentrations. Notably, UDE application, containing 100 µM γ-EC, significantly reduced NO generation compared to the control (Fig. 6). In terms of IL-1β production, LPS-stimulated BV-2 cells pretreated with 100 and 1000 µM of γ-EC or GSH showed a significant reduction compared to the control (Fig. 7). UDE, containing 10 and 100 µM γ-EC, also led to a significant decrease in IL-1β production compared to the control (Fig. 7). When examining TNF-α production in LPS-stimulated BV-2 cells, a significant reduction was observed in cells pretreated with 100 and 1000 µM γ-EC or 100 µM GSH compared to the control (Fig. 8). Interestingly, the application of UDE, containing 100 µM γ-EC, resulted in a significant decrease in TNF-α production compared to the control (Fig. 8). Collectively, these findings suggest the potential anti-neuroinflammatory effects of γ-EC, GSH, and UDE.

### γ-EC and GSH standards and UDE augmented BV-2 intracellular GSH content

**Figure 9:**
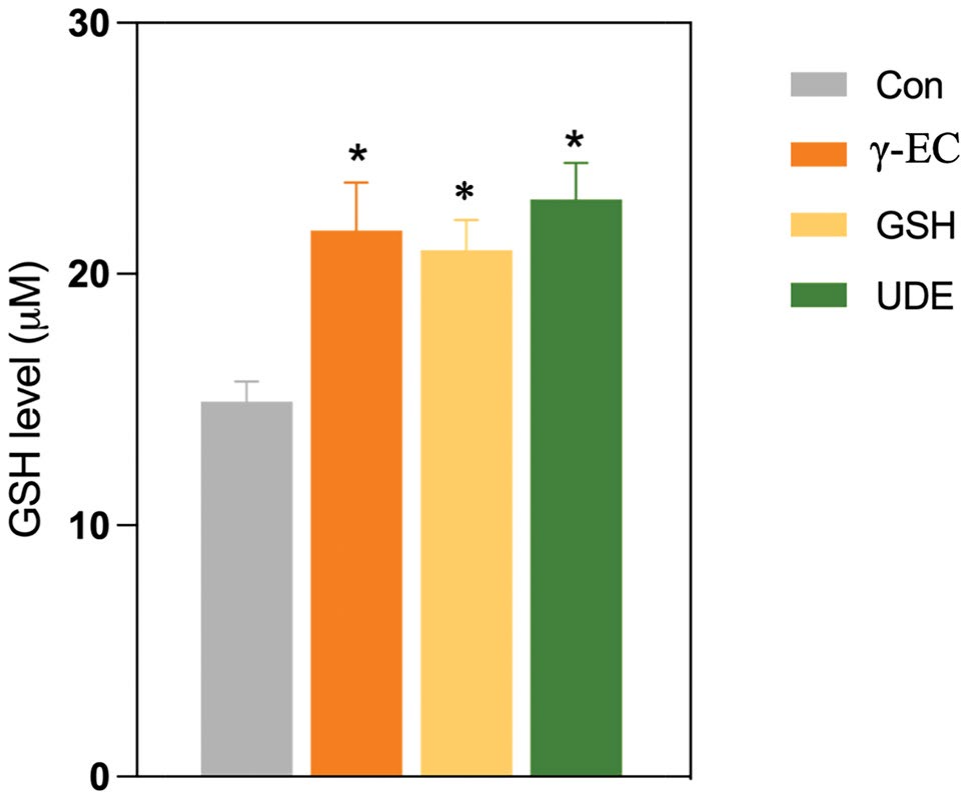
Effect of γ-glutamylcysteine (γ-EC) and glutathione (GSH) standards and the unripe durian fruit pulp extract (UDE) on intracellular GSH content of BV-2 cells. The levels of intracellular GSH were measured following a 4 h incubation with 100 µM of γ-EC, GSH, or UDE containing 100 µM γ-EC. Data are expressed as the mean ± standard error of the mean (SEM) (n = 3). *p < 0.05 vs Con (control; cells without incubation).

In our previous observations, we noted the anti-neuroinflammatory effect of a 4 h incubation with 100 µM of γ-EC, GSH, or UDE containing 100 µM γ-EC against the generation of proinflammatory cytokines induced by LPS. We then investigated whether this protective effect was due to increased intracellular GSH levels. We observed an increase in intracellular GSH content in cells treated with 100 µM of γ-EC, GSH, or UDE containing 100 µM γ-EC, compared to the untreated control cells (Fig. 9). These findings suggest that GSH acts as an anti-inflammatory agent by downregulating the release of proinflammatory cytokines.

## Discussion

Uncontrolled production of ROS or a decrease in antioxidant activity can lead to a harmful condition known as oxidative stress. Neurodegenerative disorders, including Alzheimer’s and Parkinson’s diseases, are often linked to neuronal cell death induced by oxidative stress. Oxidative stress and depletion of cellular GSH are considered key factors in the onset and progression of various chronic and age-related disorders. Therefore, methods to increase endogenous GSH levels in neurodegeneration associated with oxidative stress are of significant therapeutic interest. Plant-based remedies, which are rich in antioxidants and typically have minimal side effects, are currently being investigated for their potential benefits in this area.

Durian fruit pulp is a notable reservoir of GSH and γ-EC, although their concentrations fluctuate during distinct fruit developmental and ripening stages. As highlighted by Pinsorn et al.^28^, GSH and γ-EC attain peak levels at both the unripe and ripe stages of the fruit in the ‘Monthong’ cultivar. Given the heightened presence of volatile sulfur compounds in ripe durian pulp^28^, known for their contributions to the unpleasant roasty and onion-like odor^36^, our primary objective in this investigation was to explore the protective effects of UDE containing no volatile sulfur compounds against oxidative stress-induced neurotoxicity and neuroinflammation in human cells. Cell line, an established model for investigating the pharmacological properties of herbal extracts^37^, was employed in this study to assess the neuroprotective potential of UDE against H_2_O_2_-induced neurotoxicity in SH-SY5Y cells. The choice of SH-SY5Y cells was guided by their status as a permanently established neuronal cell line, valued for their human origin, possession of catecholaminergic neuronal properties, and ease of maintenance^38^. H_2_O_2_ functioning as an oxidizing agent, induces oxidative stress by heightening ROS production. The induction of cytotoxicity by H_2_O_2_ is a widely adopted approach to explore the potential neuroprotective and antioxidant effects of natural products^39^. Neural cells exposed to H_2_O_2_ may undergo apoptotic-like delayed death or necrosis^40^. Additionally, we examined UDE’s anti-neuroinflammatory effect by downregulating proinflammatory cytokines using LPS-stimulated BV-2 cells. Neuroinflammation, a pathological process in the central nervous system, plays a pivotal role in various acute and chronic brain diseases^41^. Glial cells, particularly microglia, are key contributors to neuroinflammation, with microglial activation leading to the production of proinflammatory cytokines like NO, IL-1β, and TNF-α^42^. Inhibiting microglial activation stands out as a potential strategy for treating neurological diseases associated with neuroinflammation. The BV-2 cell line, representing microglia, serves as a well-established in vitro model for studying microglia-induced neuroinflammation^43^. LPS, a glycolipid, does not directly influence BV-2 microglial cell activity (consistent with our observation; Supplementary Fig. S1) but expedites the release of proinflammatory cytokines^44^.

In this investigation, the application of γ-EC and GSH standards, as well as UDE, revealed no cytotoxic effects on SH-SY5Y cells (Fig. 1) or BV-2 cells (Fig. 5), with the exception of exposure to 10,000 µM concentrations of the standards and UDE containing 1000 µM γ-EC. Under these circumstances, a significant reduction in cell viability was observed (Figs. 1 and 5). This observed cytotoxicity may be attributed to the elevated uptake of GSH and/or γ-EC, potentially disturbing GSH homeostasis. The maintenance of optimal reduced/oxidized ratios within cell compartments is crucial for healthy cellular redox^45^, and disruption caused by high concentrations of these substances could explain the observed adverse effects on cell viability. Additionally, the utilization of UDE containing 1000 µM γ-EC resulted in a negative impact on cell viability, whereas treatment with 1000 µM γ-EC alone did not exhibit any toxic effects. This discrepancy could be attributed to the presence of various metabolites, including GSH in UDE, which may have contributed to cytotoxicity. We then assessed the neuroprotective effects of both standards and UDE on H_2_O_2_-induced neurotoxicity in SH-SY5Y cells. A 4 h incubation with 100 µM γ-EC or GSH before H_2_O_2_ treatment significantly enhanced cell viability (Fig. 2) across various incubation periods. Furthermore, under H_2_O_2_ stimulation, cells pretreated with UDE containing 100 µM γ-EC exhibited a significant increase in cell viability compared to H_2_O_2_-induced cells without pretreatment (Fig. 3). These results strongly indicate the neuroprotective effects of both standards and UDE against oxidative damage induced by H_2_O_2_. In parallel, we scrutinized the anti-neuroinflammatory effects of both standard compounds and UDE by examining their impact on the downregulation of proinflammatory cytokines in LPS-stimulated BV-2 cells. In this context, pretreatment with 100 µM γ-EC or the UDE containing 100 µM γ-EC significantly decreased NO production following LPS exposure. However, the application of GSH did not exhibit any noticeable effect on NO production (Fig. 6), which could be the subject of further investigation. Furthermore, cells incubated with 100 µM γ-EC, GSH, or UDE containing 100 µM γ-EC demonstrated significantly lower levels of LPS-induced IL-1β (Fig. 7) and TNF-α (Fig. 8) than the control group. In summary, when exposed to H_2_O_2_ or LPS induction, pretreatment with either 100 µM γ-EC or the UDE containing 100 µM γ-EC demonstrates a neuroprotective effect that is primarily attributed to the provision of the limiting substrate (γ-EC) and the promotion of GSH synthesis. Consequently, intracellular GSH levels were elevated (Figs. 4 and 9), leading to a reduction in the levels of proinflammatory cytokines (Figs. 6, 7, and 8).

Our findings align with previous research demonstrating the protective effects of γ-EC treatment against oxidative damage and inflammation, both in vitro (including astrocytes and neurons^19^, astrocytes^23^, and RAW264.7 cells^18^) and in vivo^19^. This protection is primarily attributed to the elevation of intracellular GSH levels. GSH exhibits multiple antioxidant actions, involving direct conjugation with free radicals such as ROS and reactive nitrogen species^46^ and enzyme-mediated neutralization of free radicals, including GSH peroxidase (GPx), which detoxifies peroxides with GSH acting as an electron donor^47^.

In this study, we report, for the first time, the presence of γ-EC in UDE as a potential neuroprotective biomarker associated with the augmentation of intracellular GSH levels. It’s noteworthy that previous studies extensively documented the pharmacological properties of durian fruit pulp extract, including its antiproliferative^30–34^, neuroprotective^35^, and antidiabetic^32^ activities on human cell lines. Despite the comprehensive nature of these studies, none have identified specific biomarkers within the durian extract until now.

Supplementation with GSH is generally ineffective at increasing cellular glutathione levels because most human cells cannot uptake extracellular GSH due to the lack of the membrane-bound ectoenzyme γ-glutamyltranspeptidase (γ-GT) or due to an unfavorable concentration gradient across the cell membrane^10,16^. In contrast, γ-EC can be readily absorbed by many cell types without thermodynamic limitations on transmembrane transport^19,24,48^. With cytosolic concentrations of γ-EC around 7 μM^49^, any passive influx of exogenous γ-EC, unlike GSH, would be directed into the cell^50^. Inside the cell, γ-EC quickly combines with glycine to form GSH, facilitated by the enzyme GSS. A clinical study involving healthy, non-fasting adults demonstrated that oral administration of γ-EC significantly increased lymphocyte GSH levels, indicating its systemic bioavailability^24^. Additionally, Le et al.^19^ showed that a single intravenous injection of γ-EC in mice significantly raised GSH levels in the brain. Importantly, the γ-glutamyl bond in γ-EC is resistant to degradation by proteases and amino proteases^51^. These findings collectively suggest that γ-EC is bioavailable and may serve as a potential therapeutic agent. However, further in vivo studies, including animal and human trials, are necessary to confirm the bioavailability of γ-EC in UDE and its potential therapeutic effects.

Maintaining the optimal balance between reduced and oxidized GSH within cell compartments is essential for preserving cellular redox homeostasis^45^. Any disruption caused by high concentrations of γ-EC supplementation could lead to adverse effects on cell viability (as observed in our study for the application of γ-EC standard at 10,000 µM (Figs. 1 and 5). In addition, we observed the cytotoxic effects of UDE containing 1000 µM γ-EC (Figs. 1 and 5). In the study conducted by Zarka and Bridge^24^, two different doses of γ-EC (2 and 4 g) were administered. To better understand the pharmacokinetics of UDE and its potential to enhance intracellular GSH levels, it is important to conduct in vivo trials that involve administering different doses of UDE at various time intervals.

Of note, durian fruit pulp contains phenolic compounds^52^. These phenolics found in UDE could exert an antioxidative protective effect against H_2_O_2_-induced oxidative stress and neuroinflammation. Previous studies have well documented the mechanisms by which phenolics protected the cells against H_2_O_2_-induced injury, including stimulating the activity of antioxidant enzymes (superoxide dismutase (SOD), glutathione peroxidase (GSH) and catalase (CAT)), reducing intracellular ROS level and lipid peroxidation, and improving mitochondrial function (via alleviating apoptosis and loss of mitochondrial membrane)^53–56^. In addition, phenolics exhibit their anti-inflammatory effects through the suppression of pro-inflammatory mediators and cytokines expression^57,58^.

Durian fruit pulp is recognized for its rich content of GSH and γ-EC, making it a valuable natural source of these compounds. The potential of developing durian pulp extract as a functional dietary supplement for preventing and protecting against neurodegeneration is promising. In Southeast Asian durian orchards, a substantial amount of organic agricultural waste is produced annually. In 2020 alone, approximately one million tons were generated in Thailand, according to data from FAOSTAT, as reported in Khaksar et al.^59^. This organic waste includes rejected fruits, providing an environmentally friendly opportunity for efficient extract production.

To summarize, this study reports the neuroprotective properties of durian fruit pulp extract. It demonstrated protection against H_2_O_2_-induced neurotoxicity in SH-SY5Y cells (a human neuroblastoma cell line) and neuroinflammation in LPS-stimulated BV-2 cells (a microglial cell line). The protective effects were observed through the promotion of GSH synthesis, which led to an increase in intracellular GSH content and a decrease in proinflammatory cytokines. To fully explore the potential health benefits of durian fruit pulp extract as a functional supplement and to investigate the long-term effects of γ-EC as a potential neuroprotective biomarker, further in vivo studies, including animal and human trials, are recommended.

## Materials and methods

### Plant materials

*After obtaining required permissions from the local authority and the orchard owner, unripe durian fruits (*cv. ‘Monthong’ at ~ 110 days after anthesis) *were collected* from a commercial durian orchard in Nonthaburi province, Thailand. The fruits were identified and authenticated (voucher specimen No. OV003-10) by with the kind assistance of a botanist from the Department of Biochemi*stry, Faculty of Science, Chulalongkorn University.*

### Extraction

Unripe durian fruit pulps were freeze-dried and ground into a fine powder. A 2-g sample of this powder was macerated with 100 mL of 50% ethanol. The mixture was then shaken vigorously at 15 °C for 2 h at 250 rpm, followed by centrifugation at 12,000 × *g* for 15 min. The supernatants were collected and filtered through 0.45-µm membrane filters. These filtered extracts were concentrated using a rotary evaporator under reduced pressure at 40 °C. The extracts were then freeze-dried for 3 days. The yield of the extract was 10.25% W/W.

### Determination of γ-EC and GSH levels using high-performance liquid chromatography (HPLC)

The dried extract (1 mg) underwent homogenization in 40 µl of 0.1 M HCl. The resulting mixture was combined with 80 μl of 25 μM N-acetylcysteine (internal standard), reduced using 20 μl of 1.58 M N-ethylmorpholine and 8 μl of 25 mM tris-(2-carboxyethy l) phosphine, and labeled with 8 μl of 30 mM monobromobimane. To halt the labeling reaction, a 10-μl aliquot of 100% acetic acid was added. The solution obtained was then centrifuged at 1000 × *g* for 10 min at 4 °C, and the supernatant was filtered through a 0.2-μm nylon syringe filter for subsequent HPLC analysis^28^.

The profiling of γ-EC and GSH in the extract was conducted using an HPLC system (Shimadzu scientific, Japan) with an autosampler. The analytical column (Phenomenex Corporate, CA, USA) utilized was a C_18_ column (5 μm, 4.6 × 250 mm) set at 45 °C, with a flow rate of 1 mL/min and an injection volume of 5 µl. Mobile phase A comprised a mixture of 40 mM sodium acetate and 17% methanol, while mobile phase B was 100% methanol. Fluorescence detection occurred with excitation at 380 nm and emission at 480 nm. The retention times for γ-EC and GSH were 6.84 and 7.74 min, respectively^28,60,61^. The average amounts of γ-EC and GSH in the durian extract were 26.3 ± 1.44 and 6.8 ± 0.52 μM, respectively (n = 3). Supplementary Fig. S2 displays chromatograms of the standards and UDE.

### Cell culture and maintenance

SH-SY5Y cells were cultured in Dulbecco’s modified eagle medium (DMEM-F12), while BV-2 microglial cells were cultivated in DMEM. Both types of media were enriched with 10% fetal bovine serum and 0.1% penicillin and streptomycin. The cells were kept in a humidified environment with 5% CO_2_ at a temperature of 37 °C.

### Effects of γ-EC and GSH standards and UDE on SH-SY5Y cell viability

The cytotoxicity of γ-EC and GSH standards, as well as UDE, was evaluated in SH-SY5Y cells using the resazurin assay. SH-SY5Y cells were placed in 96-well plates at a density of 5 × 10^4^ cells/well. The cells were exposed to varying concentrations (1, 10, 100, 1000, 10,000 μM) of the standards and UDE containing γ-EC at 1, 10, 100, and 1000 μM at 37 °C for a duration of 24 h. A resazurin solution (0.01 mg/ml) was added 4 h prior to the measurement. The fluorescence intensity was then measured at 530–590 nm using a CLARIOstar® microplate reader from BMG LABTECH, Germany.

### Protective effects of γ-EC and GSH standards and UDE on H_2_O_2_-induced cell injury in SH-SY5Y cells

The protective effects of standards and UDE against H_2_O_2_-induced cytotoxicity in SH-SY5Y cells were evaluated. The cells were seeded in 96-well plates at a density of 5 × 10^4^ cells/well and incubated with varying concentrations (100, 1000, and 10,000 µM) of the compounds for 2, 4, and 6 h. After incubation, the media were removed, and 300 µM of H_2_O_2_ was added to the wells followed by 6 h incubation. A resazurin solution was added 4 h before the measurement. Fluorescence intensity was measured at 530–590 nm using a CLARIOstar® microplate reader (CLARIOstar®, BMG LABTECH, Germany). The optimal incubation period (4 h) was used to assess the effect of UDE. Briefly, SH-SY5Y cells were incubated with UDE containing 1, 10, and 100 µM γ-EC for 4 h, then treated with H_2_O_2_. The cells were incubated for another 6 h, and cell viability was measured as previously described.

### Effects of γ-EC and GSH standards and UDE on BV-2 cell viability

The cytotoxicity of γ-EC and GSH standards, as well as UDE, was evaluated in BV-2 cells using a resazurin assay. BV-2 cells were placed in 96-well plates at a density of 2 × 10^4^ cells/well. The cells were exposed to varying concentrations (1, 10, 100, 1000, 10,000 μM) of the standards and UDE containing γ-EC at 1, 10, 100, and 1000 μM at 37 °C for a duration of 24 h. A resazurin solution (0.01 mg/ml) was added 4 h before the measurement. Fluorescence intensity was measured at 530–590 nm using a CLARIOstar® microplate reader (CLARIOstar®, BMG LABTECH, Germany).

### Effects of γ-EC and GSH standards and UDE on LPS-stimulated proinflammatory cytokines released by BV-2 cells

#### Measurement of nitric oxide (NO)

An NO assay was utilized to measure NO production by LPS-stimulated BV-2 cells. The cells were seeded in 24-well plates at a density of 2 × 10^5^ cells/well. They were then treated with non-toxic doses (1, 10, 100, 1000 µM) of standards and UDE containing γ-EC at 0.1, 1, 10, and 100 µM for 4 h. Following this, the cells were treated with 2 µg/mL LPS for 20 h. Post-treatment, the media were collected. A 100 µl sample of each collected medium was added to a 96-well plate, followed by the addition of 100 µl of modified Griess reagent (Sigma–Aldrich St. Louis, MO, USA). The plate was incubated in the dark for 5 min. Sodium nitrite (1.56, 3.12, 6.25, 12.5, 25, 50, 100 µM) was used as a standard for this measurement. The absorbance was measured at 530 nm using a CLARIOstar® microplate reader (CLARIOstar®, BMG LABTECH, Germany). The 24-well plates were incubated with the resazurin solution for 4 h to measure cell viability.

#### Measurement of interleukin (IL)-1β and tumor necrosis factor-α (TNF-α)

The inflammatory cytokines, TNF-α, and IL-1β were quantified using ELISA kits (430915 and 432615; Thermo Scientific, Rockford, IL, USA). BV-2 cells were seeded in 24-well plates overnight at a density of 2 × 10^5^ cells/well. Subsequently, the cells were treated with various doses (100 and 1000 µM) of standards and UDE containing γ-EC at 10 and 100 µM for 4 h. Following this treatment, the cells were exposed to 2 µg/mL of LPS for 20 h. The supernatant media were collected for measuring cytokine release. The ELISA plates were initially coated with the capture antibody overnight at 4 °C. Afterward, the plates underwent washing with tris-buffered saline containing 0.005% Tween-20 (TBST) four times. The assay diluent was then added to the plates and incubated for 1 h on a shaker to block nonspecific binding. Following the blocking step, the plates were washed four times with the washing reagent and incubated with 100 µl of the standards and samples for 2 h at room temperature with shaking. Subsequently, the plates were washed four times, and the detection antibody was added for incubation at room temperature for 1 h on a shaker. Avidin horseradish peroxidase (avidin-HRP) was introduced for 30 min with shaking, followed by additional washing steps. The plates were then incubated with the 3,3′,5,5′-tetramethylbenzidine (TMB) substrate solution for 15 min in the dark. The reaction was halted using a 2 N H_2_SO_4_ solution, and the absorbance was measured at 450 nm using a CLARIOstar® microplate reader (CLARIOstar®, BMG LABTECH, Germany).

#### Measurement of intracellular GSH levels

Intracellular GSH levels in SH-SY5Y and BV-2 cells were assessed using a GSH assay kit (CS0260, Sigma, Merck KGaA, Darmstadt, Germany). SH-SY5Y and BV-2 cells were grown in 6-well plates at densities of 1 × 10^6^ and 5 × 10^5^ cells/well, respectively. The cells were then incubated with 100 µM of γ-EC and GSH standards and the extract containing 100 µM γ-EC for 4 h. The cells were washed and resuspended in ice-cooled phosphate-buffered saline in centrifuge tubes. After centrifugation at 600 × g for 10 min, the supernatants were removed. The cell pellets were homogenized with 5% sulfosalicylic acid and centrifuged at 10,000 × *g* for 10 min. The supernatants were collected and incubated with the working mixture for 5 min. NADPH solution was added to the plates, and the absorbance was measured at 412 nm using a CLARIOstar® microplate reader (CLARIOstar®, BMG LABTECH, Germany).

### Statistical analysis

The data were analyzed using GraphPad Prism software (version 8.0.2, GraphPad Software, San Diego, California, USA). The results are presented as the mean ± standard error of the mean (SEM). Statistical evaluation was conducted by one-way ANOVA followed by a Tukey’s post hoc test. A *p*-value less than 0.05 was considered statistically significant.

### Supplementary Information

Supplementary Figures.

## Data Availability

Data available within the article or its supplementary materials.
