# Supplementary material for: Durian fruit pulp extract enhances intracellular glutathione levels, mitigating oxidative stress and inflammation for neuroprotection
Source: Sci Rep. 2024 Jul 2;14:15153. doi: 10.1038/s41598-024-65219-6 (PMC11220076; doi:10.1038/s41598-024-65219-6)
Supplement: Supplementary file 1 — Supplementary Figures. [file 41598_2024_65219_MOESM1_ESM.docx]

**Durian fruit pulp extract enhances intracellular glutathione levels, mitigating oxidative stress and inflammation for neuroprotection**

Gholamreza Khaksar^1^, Su Lwin Lwin Myint^2^, Hasriadi^3^, Pasarapa Towiwat^3,4^, Supaart Sirikantaramas^1,5,*^, Ratchanee Rodsiri^2,4,*^

^1^Center of Excellence in Molecular Crop, Department of Biochemistry, Faculty of Science, Chulalongkorn University, 254 Phayathai Road, Bangkok 10330, Thailand

^2^Preclinical Toxicity and Efficacy Assessment of Medicines and Chemicals Research Unit, Chulalongkorn University 10330, Thailand

^3^Animal Models of Chronic Inflammation-associated Diseases for Drug Discovery Research Unit, Chulalongkorn University, Bangkok 10330, Thailand

^4^Department of Pharmacology and Physiology, Faculty of Pharmaceutical Sciences, Chulalongkorn University 10330, Thailand

^5^Omics Sciences and Bioinformatics Center, Chulalongkorn University, 254 Phayathai Road, Bangkok 10330, Thailand

*Corresponding authors
Supaart Sirikantaramas [supaart.s@chula.ac.th](mailto:supaart.s@chula.ac.th), Ratchanee Rodsiri [ratchanee.r@pharm.chula.ac.th](mailto:ratchanee.r@pharm.chula.ac.th)

**Supplementary material for this submission is available as follows:**

Supplementary Figure S1

Supplementary Figure S2


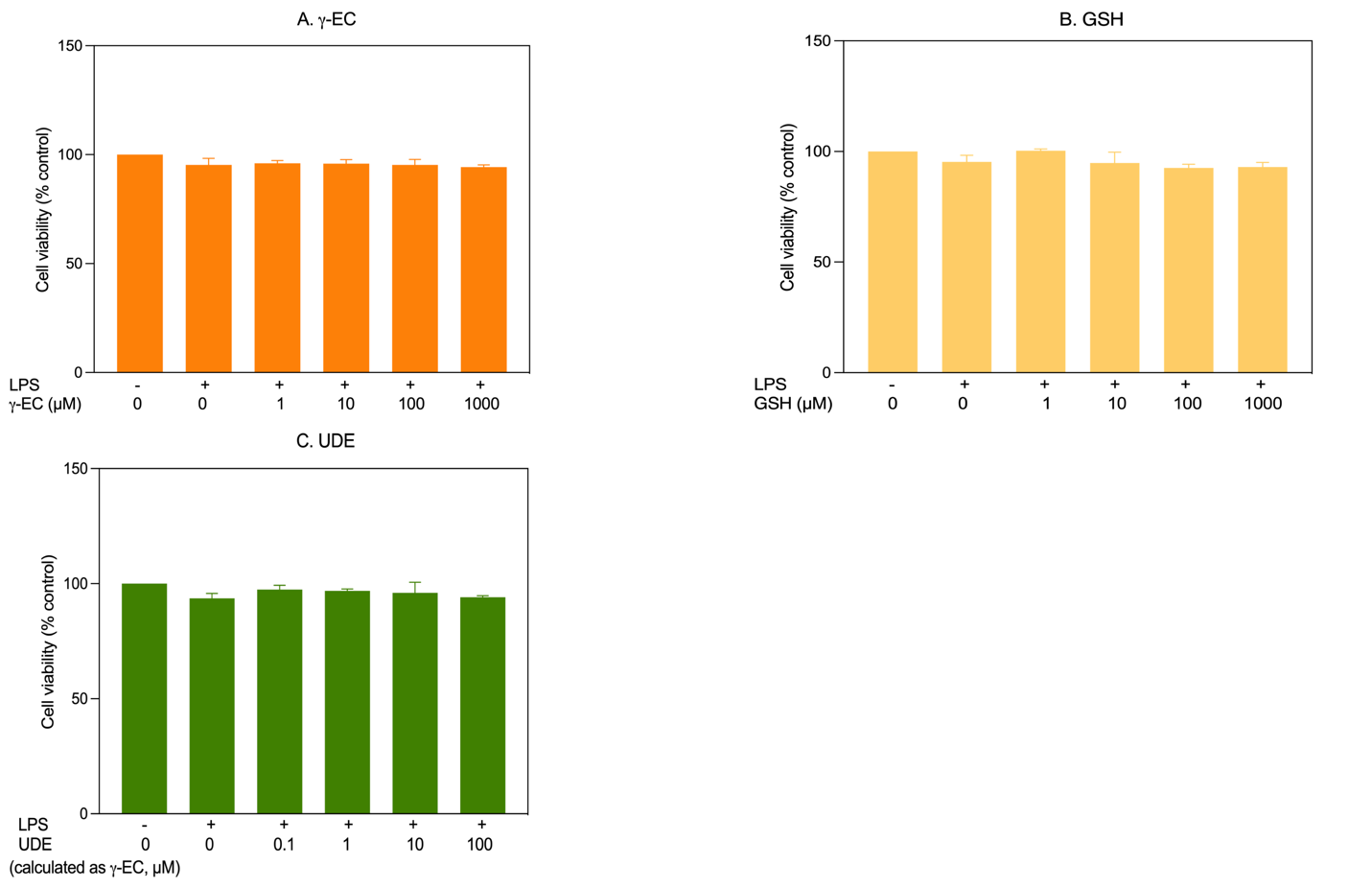


**Supplementary Figure S1.** Cell viability of lipopolysaccharide (LPS)-stimulated BV-2 cells incubated with varying concentrations of γ-glutamylcysteine (γ-EC) **(A)**, glutathione (GSH) **(B)**, or unripe durian fruit pulp extract (UDE) containing different γ-EC concentrations **(C)**. Data are presented as the mean ± standard error of the mean (SEM) (n = 3). No significant differences were observed.


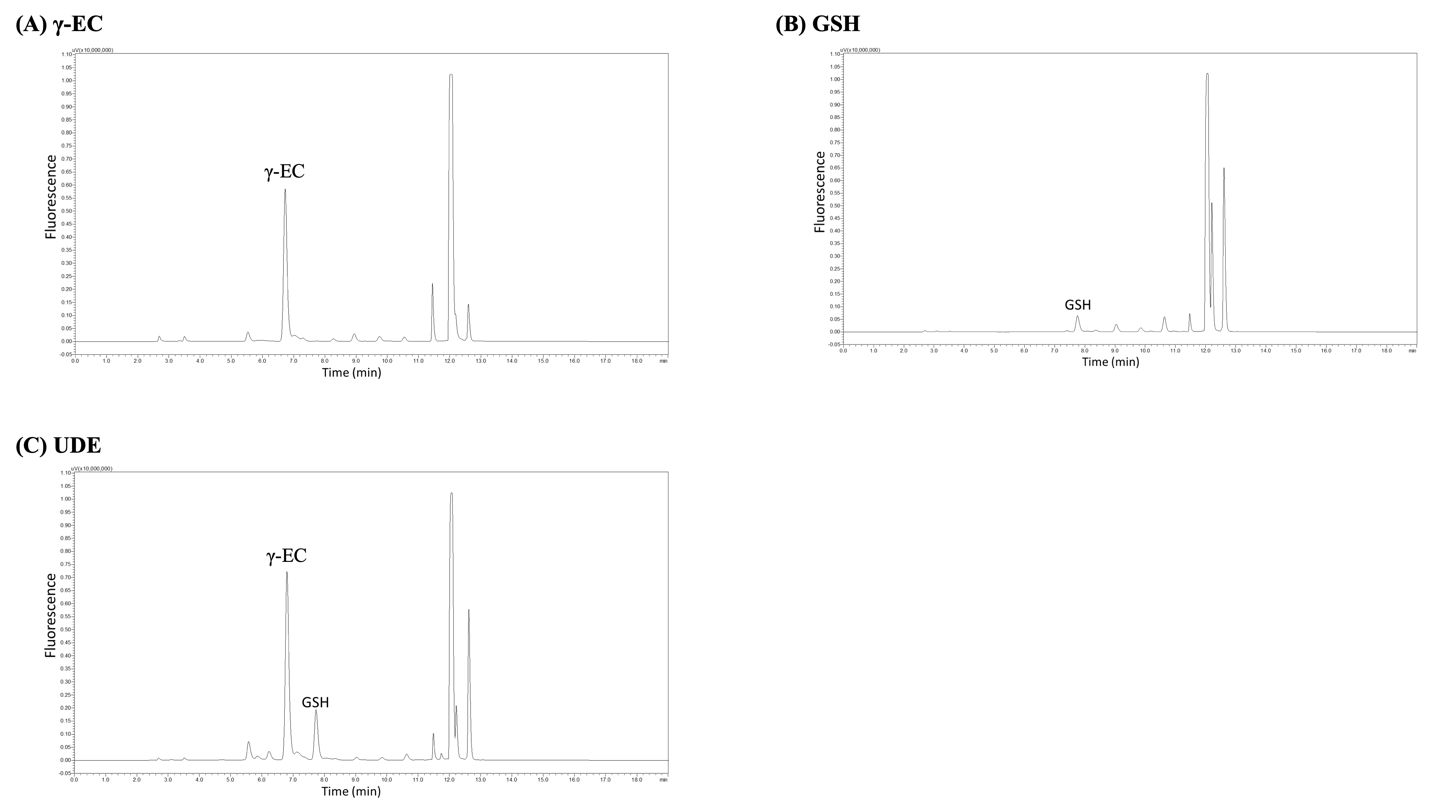


**Supplementary Figure S2.** Chromatograms of the γ-glutamylcysteine (γ-EC) **(A)** and glutathione (GSH) **(B)** standards and the unripe durian fruit pulp extract (UDE) **(C)** are presented.
